# Nestin expression in osteosarcomas and derivation of nestin/CD133 positive osteosarcoma cell lines

**DOI:** 10.1186/1471-2407-8-300

**Published:** 2008-10-16

**Authors:** Renata Veselska, Marketa Hermanova, Tomas Loja, Petr Chlapek, Iva Zambo, Karel Vesely, Karel Zitterbart, Jaroslav Sterba

**Affiliations:** 1 Laboratory of Tumor Biology and Genetics, Institute of Experimental Biology, School of Science, Masaryk University, Kotlarska 2, 611 37 Brno, Czech Republic; 2 Department of Pediatric Oncology, University Hospital Brno, Cernopolni 9, 613 00 Brno, Czech Republic; 3 1st Institute of Pathologic Anatomy, St. Anne's University Hospital, Pekarska 53, 656 91 Brno, Czech Republic; 4 Institute of Pathology, University Hospital Brno, Jihlavska 20, 625 00 Brno, Czech Republic

## Abstract

**Background:**

Nestin was originally identified as a class VI intermediate filament protein that is expressed in stem cells and progenitor cells in the mammalian CNS during development. This protein is replaced in the adult organism by other intermediate filament proteins; however, nestin may be re-expressed under certain pathological conditions such as ischemia, inflammation, brain injury, and neoplastic transformation. Nestin has been detected in many kinds of tumors, especially in tumors derived from the CNS. Co-expression of nestin and the CD133 surface molecule is considered to be a marker for cancer stem cells in neurogenic tumors. Our work was aimed at a detailed study of nestin expression in osteosarcomas and osteosarcoma-derived cell lines.

**Methods:**

Using immunodetection methods, we examined nestin in tumor tissue samples from 18 patients with osteosarcomas. We also successfully established permanent cell lines from the tumor tissue of 4 patients and immunodetection of nestin and CD133 was performed on these cell lines.

**Results:**

Nestin-positive tumor cells were immunohistochemically detected in all of the examined osteosarcomas, but the proportion of these cells that were positively stained as well as the intensity of staining varied. Nestin-positive cells were rarely observed in 2 tumor samples, and the remaining 16 tumor samples showed various nestin expression patterns ranging from very sporadic occurrence to an overwhelming proportion of cells with strong positive staining. Three of the established osteosarcoma cell lines were demonstrated to be nestin-positive, and only one cell line showed no expression of nestin; this finding corresponds with the rare occurrence of nestin-positive cells in the respective tumor sample. Moreover, three of these osteosarcoma cell lines were undoubtedly proven to be Nes+/CD133+.

**Conclusion:**

Our results represent the first evidence of nestin expression in osteosarcomas and suggest the possible occurrence of cells with a stem-like phenotype in these tumors.

## Background

Nestin, a neural stem cell protein, was identified as a class VI intermediate filament protein. The molecule consists of 1,618 amino acids and its molecular weight is 176 kDa [1-3]; the nestin gene contains 4 exons and 3 introns [4]. Nestin expression has been shown in proliferating neuroepithelium during the development of the mammalian CNS, as well as in both human and rodent neural stem cells in vivo [5-7]. Nestin was also expressed in various immortalized mammalian stem cell lines and precursor cell lines [8]. In the adult CNS, nestin is detectable only in the stem cells of the subventricular zone and in the choroid plexus [7]. Re-expression of downregulated nestin was reported in reactive astrocytes following certain types of brain injuries, as well as in reactive astrocytes and endothelial cells in cerebral abscesses [9,10].

Immunodetection has shown that nestin is expressed in many kinds of tumors, especially in tumors derived from CNS (e.g., central neurocytomas, gangliogliomas, ependymomas, pilocytic astrocytomas, high-grade gliomas including glioblastoma multiforme), and embryonal tumors originating from the CNS (primitive neuroectodermal tumors – PNETs, medulloblastomas, and medulloepitheliomas) [5,11-24]. Nevertheless, nestin expression was also detected in rhabdomyosarcomas [25], gastrointestinal stromal tumors (GISTs) [26-29], malignant melanomas [30,31], hepatocellular carcinomas, cervical carcinomas, and ovarian carcinomas [32]. Its occurrence in tumor cells is not limited to the cytoplasm only; nestin localization in cell nuclei was clearly confirmed in some neuroblastoma and glioblastoma cell lines [33,34]. Coexpression of nestin and CD133 (also known as prominin-1) is considered to be a marker for cancer stem cells (CSCs); this fact was experimentally proven in glioblastoma multiforme and malignant melanoma [31,35,36].

The present study was aimed at the examination of nestin in tumor tissue samples taken from patients with osteosarcomas and in cell lines derived from these tumors using immunohistochemistry and immunofluorescence. Nestin-positive (Nes+) tumor cells were detected in all of them, but the proportion of the cells that expressed nestin as well as the intensity of the staining varied from a rare occurrence of Nes+ cells to an overwhelming proportion of cells with high nestin expression. We also successfully derived permanent cell lines from the tumor tissues of four patients with osteosarcoma and three of these cell lines were undoubtedly proven to be Nes+/CD133+. Our results represent the first evidence of nestin expression in osteosarcomas and suggest the possible occurrence of cells with stem-like phenotype in these tumors.

## Methods

### Tumor samples

Eighteen samples of primary untreated high-grade osteosarcoma of bone (17 conventional osteosarcomas: 15 osteoblastic and 2 chondroblastic, and 1 telangiectatic osteosarcoma; 9 males, 9 females; age range: 8–57 years old, mean 21 years old) were included in this study. Formalin-fixed and paraffin-embedded surgical samples of neoplastic tissues were retrieved from the files of the Department of Pathology, University Hospital Brno, Czech Republic, and of the 1^st ^Department of Pathologic Anatomy, St. Anne's University Hospital, Brno, Czech Republic. The histologic sections stained with H-E were reviewed by three pathologists in total and each individual tumor sample by two of them (MH and IZ; or MH and KV), and representative tissue blocks were selected for immunohistochemical analysis. Fifteen samples were not ossified and were not subjected to decalcification and three cases were decalcified using 8% hydrochloride acid-ferric chloride solution, as indicated in the Table 1. To obtain cell cultures, biopsy samples were taken from 4 patients surgically treated for osteosarcoma. Written informed consent was obtained from each participant before entering into this study. The samples for cell cultures were coded and processed in the laboratory in an anonymous manner. The Research Ethics Committee of the University Hospital Brno approved the study protocol.

### Immunohistochemistry

Immunohistochemical detection of nestin was performed on 4 μm thick tissue sections applied to positively-charged slides. The sections were deparaffinized in xylene and rehydrated through a graded alcohol series. Antigen retrieval was performed in the lab microwave (Milestone) by heating the sections in citrate buffer at pH 6.0 for 20 min at 98°C. Endogenous peroxidase activity was quenched in 3% hydrogen peroxide in methanol for 10 minutes. Tissue sections were incubated overnight at 4°C with a mouse monoclonal antibody to nestin (clone 10C2, dilution 1:200, Millipore, Billerica, MA, USA). A streptavidin-biotin peroxidase detection system was used in accordance with the manufacturer's instructions (Vectastain Elite Kit, Vector Laboratories, Burlingame, CA, USA); 3,3'-diaminobenzidine was used as the chromogen (DAB, Fluca, USA). Slides were counterstained with Gill's hematoxylin. Tissue sections of glioblastoma multiforme served as external positive controls; nestin-positive endothelial cells in osteosarcoma tissue samples were used as internal positive controls. Negative controls were prepared by incubating samples without the primary antibody. Evaluation of immunohistochemical results was performed using a uniform microscope and camera setting (Olympus BX51, DP70).

### Evaluation of immunohistochemistry

For nestin, cytoplasmic immunostaining was considered to be positive. The percentage of Nes+ tumor cells (TC) was counted and categorized into four levels: +/- (<2 % Nes+ TC), + (2–10 % Nes+ TC), ++ (11–50 % Nes+ TC), and +++ (51–100 % Nes+ TC). The intensity of immunostaining was classified as very weak (+/-), weak (+), medium (++), and strong (+++). The intensity of immunostaining was also evaluated in endothelial cells, which were used as an internal positive control. The slides were evaluated with a light microscope at ×400 magnification. At least 5 discrete foci of neoplastic infiltration were analyzed, and the average staining intensity and the percentage of nestin positive cells of the entire covered area were determined.

### Cell cultures

Starting with primary cultures, fresh specimens of tumor tissue were processed as described previously [34]. The primary cultures were maintained in DMEM supplemented with 20% fetal calf serum, 2 mM glutamine, and antibiotics: 100 IU/ml of penicillin and 100 μg/ml of streptomycin (all purchased from PAA Laboratories, Linz, Austria) and cultivated under standard conditions at 37°C in an atmosphere of 95% air : 5% CO_2_. Once the specimen pieces had attached, the volume of the medium was gradually increased to 5 ml over the next 48 hours. As soon as the outgrowing cells covered about 60% of the surface, they were trypsinized, diluted, and transferred into a new flask. A similar procedure was used for further subcultivations of all cell lines that were derived from the primary cultures. Altogether, 4 successfully derived osteosarcoma cell lines (OSA-1, OSA-2, OSA-3, and OSA-5) were included in this study. The basic description (gender and age) of the patients from whom the respective samples were taken is given in the Table 1. The established Saos-2 osteosarcoma cell line (ATCC No. HTB-85) and the GM-7 glioblastoma cell line [34] were used as control cell lines in this study.

### Immunofluorescence

To perform immunostaining of intracellular and cell surface antigens in these cell lines, cell suspensions at a concentration of 10^4 ^cells per ml were seeded on glass coverslips and grown under standard conditions for 24 h. Cells were then washed in PBS, fixed with 3% para-formaldehyde (Sigma) in PBS for 20 min at RT, and permeabilized with 0.2% Triton X-100 (INC Biomedicals) in PBS for 1 min at RT. For detection of the CD133 cell surface molecule, this procedure was performed without permeabilization. The cells were subsequently washed in PBS and incubated for 10 min with 2% BSA (PAA) to block nonspecific binding of the secondary antibodies. All intracellular and cell surface antigens were visualized by indirect immunofluorescence. Mouse monoclonal human-specific anti-nestin antibody (clone 10C2, dilution 1:200, Millipore), mouse monoclonal anti-vimentin antibody (clone LN-6, dilution 1:100, Sigma Chemical Co., St. Louis, MO, USA), mouse monoclonal anti-desmin antibody (clone DE-U-10, dilution 1:40, Sigma), rabbit polyclonal anti-S100 antibody (No. S2644, working concentration 20 μg.ml^-1^, Sigma), and rabbit polyclonal anti-CD133 antibody (No. ab19898, dilution 1:100, Abcam, Cambridge, UK) were used as primary antibodies. The cells were treated with primary antibodies at 37°C for 1 h and then washed three times in PBS. Corresponding secondary antibodies, i.e. anti-mouse antibodies conjugated with FITC or TRITC (Sigma) or anti-rabbit antibody conjugated with TRITC (Sigma), were applied under the same conditions. Finally, the cells were mounted onto glass slides in Vectashield mounting medium containing DAPI (Vector Laboratories, Burlingame, CA, USA). The cells were observed using an Olympus BX-61 fluorescence microscope. Micrographs were captured with a CCD camera COHU 4910 and analyzed using software Lucia 4.80 – KARYO/FISH/CGH (Laboratory Imaging, Prague, Czech Republic).

## Results

### Nestin detection in the osteosarcoma tumor tissue

**Figure 1:**
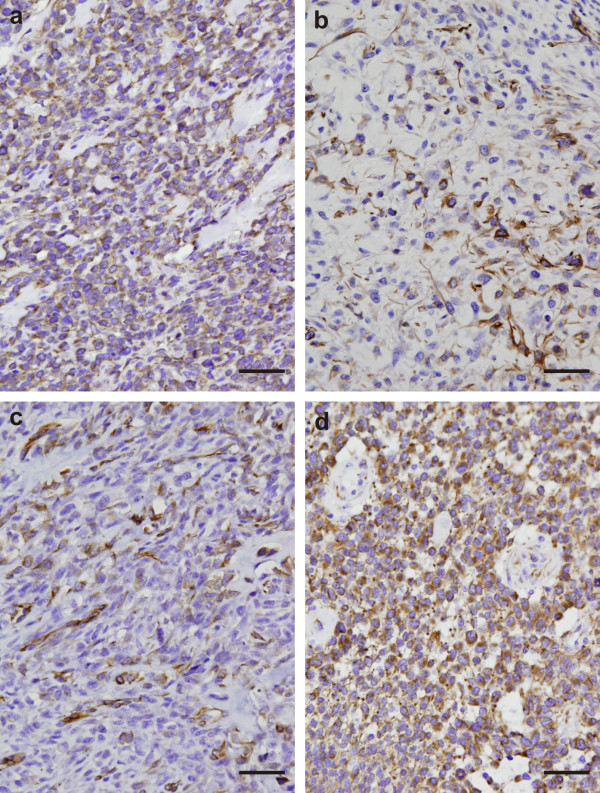
**Immunohistochemical analysis of nestin expression in conventional osteosarcomas**. Strong diffuse cytoplasmic nestin immunostaining in majority of tumor cells; sample 16 (a). Strong cytoplasmic nestin immunostaining in a subset of tumor cells; sample 11 (b). Medium to strong cytoplasmic nestin expression in a minority of tumor cells dispersedly; sample 17 (c). Glioblastoma multiforme was used as a positive control (d). Immunoperoxidase with Gill's hematoxylin counterstain. Bars, 100 μm.

The results of the immunohistochemical detection of nestin expression in osteosarcomas are summarized in Table 1. Nes+ tumor cells were found in all examined tumor samples taken from the 18 patients of both gender and various age (Figure 1a–c). Nevertheless, the frequency of these Nes+ cells ranged from the strong positivity in a significant proportion of tumor cells (Table 1, Figure 1a), through a dispersed distribution (Table 1, Figure 1b), to a sporadic occurrence of these cells (Table 1, Figure 1c). Similarly, the intensity of immunostaining for nestin varied from strong to very weak (Table 1, Figure 1a–c). All 18 tumor samples undoubtedly showed Nes+ endothelial cells.

**Table 1:** Immunohistochemical analysis of nestin expression in osteosarcomas

**Sample**	**Gender**	**Age**	**Tumor type**		**Nestin**		**Cell line**
				**% TC**	**IR TC**	**IR EC**	

1	F	8	C-OS-OB/DC	++	++	+	
2	F	14	C-OS-OB	+	+/-	+/-	
3	F	56	C-OS-OB	+	+	+/-	
4	M	57	C-OS-OB	+	+/-	+/-	
5	F	18	C-OS-OB	+	+/-	+/-	
6	M	15	C-OS-OB	++	++	+	
7	M	15	C-OS-OB	+	+	+/-	
8	F	23	C-OS-CB/DC	+	+	+	
9	M	21	C-OS-CB/DC	+	+/-	+/-	
10	F	14	C-OS-OB	+/-	+/-	+/-	
11	F	13	C-OS-OB	+	++	++	OSA-01
12	F	19	C-OS-OB	+	+	++	
13	M	21	C-OS-OB	+++	+++	++	
14	M	15	C-OS-OB	++	++	+	
15	F	28	C-OS-OB	++	+	+/-	
16	M	21	C-OS-OB	+++	+++	++	OSA-02
17	M	15	C-OS-OB	++	++	++	OSA-03
18	M	9	OS-TAE	+/-	+	+	OSA-05

Expression of nestin was examined on formalin-fixed, paraffin-embedded tissue specimens of osteosarcomas using immunohistochemistry. % TC, percentage of nestin positive tumor cells (+/-, <2 %; +, 2–10 %; ++, 11–50 %; +++, 51–100 %). IR TC, intensity of immunostaining (immunoreactivity) in tumor cells (+/-, very weak; +, weak; ++, medium; +++, strong). IR EC, intensity of immunostaining (immunoreactivity) in endothelial cells (+/-, very weak; +, weak; ++, medium; +++, strong). Tumor type: C-OS-OB, high grade conventional osteosarcoma, osteoblastic; OS-TAE, teleangiectatic osteosarcoma; C-OS-CB, high grade conventional osteosarcoma, chondroblastic; DC, decalcified.

### Expression of nestin and CD133 in osteosarcoma cell lines

**Figure 2:**
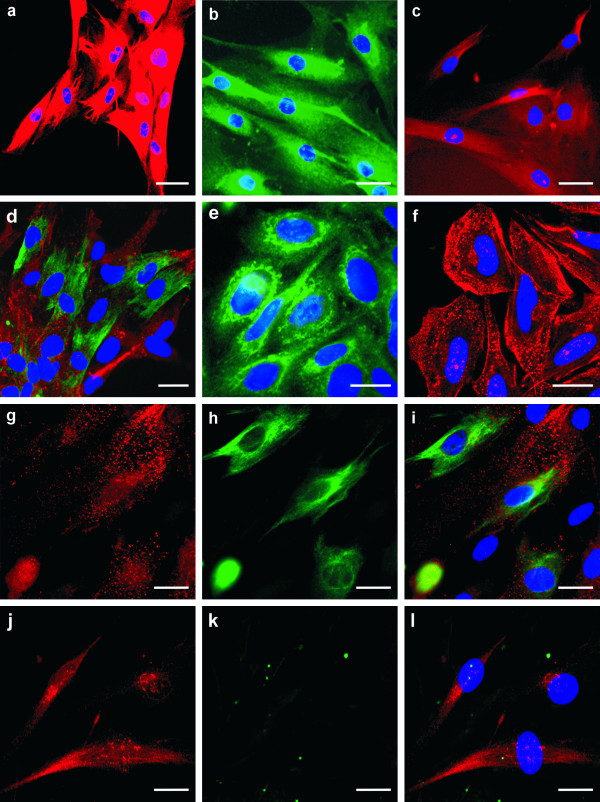
**Expression of nestin, CD133 and other intracellular proteins in the osteosarcoma cell lines**. Representative expression of vimentin, desmin, and S100 protein in the osteosarcoma cell lines: vimentin expression in OSA-03 cell line (a); desmin expression in OSA-02 cell line (b); S100 protein expression in OSA-01 cell line (c). Medium or strong expression of nestin was found in three newly derived cell lines and the distinct network of nestin-positive filaments was observable in the OSA-03 cell line (h, i). OSA-05 cell line was nestin-negative (k, l). Expression of CD133 cell surface molecule (g, i, j, l) was found in all osteosarcoma cell lines and co-expression of nestin and CD133 was confirmed using double labeling in nestin-positive cell lines (g-i). Representative double labeling for CD133 and nestin in nestin-positive OSA-03 (g-i) and nestin-negative OSA-05 (j-l) newly derived osteosarcoma cell lines. Glioblastoma GM-7 cell line labeled by the same antibodies was used as positive control (d) and control established Saos-2 osteosarcoma cell line also showed strong positivity for both nestin (e) and CD133 (f). Vimentin (a, red), S100 protein (c, red), and CD133 (d, f, g, i, j, l, red) stained by indirect immunofluorescence using TRITC-labeled secondary antibody; desmin (b, green) and nestin (d, e, h, i, k, l, green) stained by the same method using FITC-labeled secondary antibody; counterstaining with DAPI (a-f, i, l). Bars, 30 μm (a-c), 20 μm (d), 15 μm (e-l).

Immunoreactivity for nestin was examined in four newly derived osteosarcoma cell lines as described above. In the way of other intracellular markers, all of these cell lines were positive for vimentin (Figure 2a), desmin (Figure 2b), and S100 protein (Figure 2c). Results concerning the immunoreactivity for nestin, CD133 and other intracellular proteins are summarized in Table 2. Nestin was detected in three (OSA-1, OSA-2, and OSA-3) of the four examined cell lines; in the OSA-5 cell line no signal for nestin was observed (Table 2, Figure 2k–l). The OSA-3 cell line displayed a strong, even positivity for nestin and a distinct network of Nes+ filaments in the cytoplasm of individual cells (Figure 2h–i), while OSA-1 and OSA-2 cell lines showed medium intensity of fluorescence, and only the diffuse signal in the cytoplasm was observable. All newly derived cell lines also showed a strong positivity for the CD133 membrane antigen (Figure 2g,i), including the nestin-negative OSA-5 cell line (Figure 2j,l). Double-labeling of both of these antigens confirmed their appearance in the same cells (Figure 2g–i). Control Saos-2 osteosarcoma cell line also clearly showed both of nestin (Figure 2e) and CD133 (Figure 2f) positivity.

**Table 2:** Immunostaining of osteosarcoma-derived cell lines

					**Cell line**					
	**OSA-01**		**OSA-02**		**OSA-03**		**OSA-05**		**Saos-2**	
**Antigen**	**%**	**IR**	**%**	**IR**	**%**	**IR**	**%**	**IR**	**%**	**IR**

**Desmin**	+++	+	++	++	+++	++	++	+	++	+
**S-100**	+++	+	+++	+	++	+	+++	++	+++	+
**Vimentin**	++	+	++	+	+++	++	+++	+++	++	++
**Nestin**	++	++	++	++	+++	+++	-	-	+	+++
**CD133**	+++	++	+++	+++	+++	+++	+++	++	+++	+++

	**%**		**%**		**%**		**%**		**%**	

**Nestin/CD133 co-expression**	++		++		+++		-		+	

Expression of nestin, CD133 and other proteins was examined on cell cultures of osteosarcoma-derived cell lines using indirect immunofluorescence. With the exception of the nestin-negative OSA-05 cell line, all cell lines showed even immunoreactivity for the detected antigens. Percentage of positive cells in cell populations (%: -, 0%; +/-, <2 %; +, 2–10 %; ++, 11–50 %; +++, 51–100 %) and intensity of immunostaining (IR: -, none; +, weak; ++, medium; +++, strong) were evaluated. Saos-2 cell line was originally used as a control osteosarcoma cell line for the CD133 staining.

## Discussion

Our research was primarily focused on the examination of possible nestin expression in osteosarcoma tissue sections using immunohistochemistry. Obtained results clearly confirmed the presence of Nes+ tumor cells in samples taken from all patients involved in the study, although the frequency of Nes+ cells as well as the immunoreactivity varied in the individual samples. Nevertheless, our research produced the first evidence of nestin expression in the osteosarcomas.

Although the nestin was detected in many kinds of solid tumors, its expression is widely recognized as a tumor marker especially of malignancies of neuroectodermal origin [5,11-24]. For other tumors, nestin expression was still reported in GISTs [26-29], malignant melanomas [30,31], hepatocellular carcinomas, cervical carcinomas, and ovarian carcinomas [32]. However, there is only a minimum of similar findings in soft tissue sarcomas; the only one evidence of nestin was given in pediatric rhabdomyosarcomas [25] and angiosarcomas [37]. In synovial sarcomas, nestin expression was documented in approximately 10% of pediatric synovial sarcomas [38] but it was not detected in the same tumor type in another study [39]. Not very surprisingly, nestin expression was also sporadically showed in Ewing sarcomas/PNETs [39].

To verify our findings in osteosarcoma tissue sections stained by IHC, we used four cell lines that were newly derived from osteosarcomas in our laboratory. The results obtained from these cell lines by indirect immunofluorescence clearly confirmed the findings on the corresponding tissue sections. All three Nes+ cell lines showing the medium (OSA-01 and OSA-02 cell lines) or strong (OSA-03 cell line) expression of nestin were derived from the tumors containing cells with medium or strong nestin positivity (Table 1), although the frequency of these Nes+ cells varied in the individual tumors (Figure 1). The OSA-05 cell line was repeatedly demonstrated as nestin-negative during the long-term cultivation at different passages, and this cell line was derived from the only telangiectatic osteosarcoma, in which the only rare occurrence of Nes+ cells was demonstrated. The validity of these results using newly derived osteosarcoma cell lines was clearly confirmed by immunodetection of the strong nestin positivity also in the established Saos-2 cell line that was used as a control osteosarcoma cell line in our study.

In regard to the recently reported expression of CSCs marker CD133 in Saos-2 cell line cell [40], we also investigated our new osteosarcoma cell lines for this cell surface antigen. The results clearly showed the expression of this CSCs marker in all four examined cell lines. CD133 expression was strong in two of three Nes+ cell lines (OSA-02, and OSA-03) as well as in the control Saos-2 cell line, whereas medium immunoreactivity was detected in Nes+ OSA-01 cell line and in nestin-negative OSA-5 cell line.

CSCs from different histogenetic categories of tumors may vary in their pattern of specific markers; nevertheless, common phenotypic characteristics of leukemia, brain tumors, prostate cancer, lung carcinoma, malignant melanoma, head and neck squamous cell carcinoma, and breast cancer CSCs are known [41-44]. Co-expression of nestin and CD133 was previously recognized to be a typical characteristic of CSCs in CNS tumors [35], but it was found also in other tumors of ectodermal origin [31]. At present, expression of CD133 is considered to be a universal marker of CSCs also in other histogenetic types of tumors [43,45]. Our results regarding the co-expression of CD133 and nestin in newly derived osteosarcoma cell lines as well as in the established Saos-2 cell line suggest the presence of cells with CSCs characteristics also in osteosarcomas.

Biological features of CSCs and their occurrence in various types of malignancies are undoubtedly one of the topical issues in up-to-date cancer research. CSCs seem to play a key role in tumor initiation, progression, and metastasis [43,46], and they may thus represent an important target in anticancer treatment [41].

Our results concerning the first evidence of nestin expression in osteosarcomas, some previously reported findings on nestin expression in soft-tissue sarcomas [25,37,38], and particularly our detection of CSCs features in osteosarcoma-derived cell lines indicate the importance of such research with regard to the bone and soft-tissue sarcomas. Therefore, the present study is a first step to further detailed investigation of the cells exhibiting CSCs markers in osteosarcoma tumor tissue, especially in view of the clinical course of the disease.

## Conclusion

The most important result of our study is the first evidence of nestin expression in osteosarcomas. To summarize, nestin-positive tumor cells were immunohistochemically detected in all of the examined osteosarcomas, although the proportion of Nes+ cells as well as the intensity of staining varied. Three of the established osteosarcoma cell lines were demonstrated to be nestin-positive, and only one cell line showed no expression of nestin. These findings correspond with the rare occurrence of Nes+ cells in the respective tumor sample. Moreover, three of these lines as well as the control Saos-2 osteosarcoma cell line were undoubtedly proven to be Nes+/CD133+. This important finding suggests the possible occurrence of cells with a stem-like phenotype in osteosarcomas.

## Abbreviations

BSA: bovine serum albumin; C-OS-CB: high grade conventional osteosarcoma, chondroblastic; C-OS-OB: high grade conventional osteosarcoma, osteoblastic; CSCs: cancer stem cells; DAPI: 4,6-diamidino-2-phenylindol; DC: decalcified; DMEM: Dulbecco's modified Eagle's medium; EC: endothelial cells; FITC: fluorescein isothiocyanate; GISTs: gastrointestinal stromal tumors; H-E: hematoxylin-eosin; IHC: immunohistochemistry; IR: immunoreactivity; Nes+: nestin positive; OS-TAE: telangiectatic osteosarcoma; PBS: phosphate-buffered saline; PNETs: primitive neuroectodermal tumors; RT: room temperature; TC: tumor cells; TRITC: tetramethylrhodamine isothiocyanate.

## Competing interests

The authors declare that they have no competing interests.

## Authors' contributions

RV conceived the study, participated in the immunofluorescence analysis of osteosarcoma cell lines, and drafted the manuscript. MH managed the histopathological analysis of tumor samples, participated in the patient inclusion and participated in the manuscript preparation. TL and PC performed the derivation of osteosarcoma cell lines and immunofluorescence analysis of these cell lines. IZ and KV participated in the histopathological analysis of tumor samples. KZ managed the patient inclusion for this study and participated in the manuscript preparation. JS coordinated this study and participated in the patient inclusion. All authors read and approved the final manuscript.

## Pre-publication history

The pre-publication history for this paper can be accessed here:


